# Cortisol levels in blood and hair of unanesthetized grizzly bears (*Ursus arctos*) following intravenous cosyntropin injection

**DOI:** 10.1002/vms3.523

**Published:** 2021-05-12

**Authors:** Marc Cattet, David M. Janz, Luciene Kapronczai, Joy A. Erlenbach, Heiko T. Jansen, O Lynne Nelson, Charles T. Robbins, Gordon B. Stenhouse

**Affiliations:** ^1^ RGL Recovery Wildlife Health & Veterinary Services Saskatoon SK Canada; ^2^ Department of Veterinary Pathology Western College of Veterinary Medicine University of Saskatchewan Saskatoon SK Canada; ^3^ Department of Veterinary Biomedical Sciences Western College of Veterinary Medicine University of Saskatchewan Saskatoon SK Canada; ^4^ Toxicology Centre University of Saskatchewan Saskatoon SK Canada; ^5^ School of the Environment Washington State University Pullman WA USA; ^6^ Department of Integrative Physiology and Neuroscience College of Veterinary Medicine Washington State University Pullman WA USA; ^7^ Department of Veterinary Clinical Sciences College of Veterinary Medicine Washington State University Pullman WA USA; ^8^ School of Biological Sciences Washington State University Pullman WA USA; ^9^ fRI Research Hinton AB Canada; ^10^ Alberta Environment & Parks Edmonton AB Canada

**Keywords:** cosyntropin challenge, grizzly bear, hair cortisol concentration (HCC), serum cortisol concentration, *Ursus arctos*

## Abstract

Hair cortisol concentration (HCC) is being used increasingly to evaluate long‐term stress in many mammalian species. Most of the cortisol is assumed to passively diffuse from circulating blood into hair follicles and gradually accumulate in growing hair. However, our research with free‐ranging grizzly bears (*Ursus arctos*) suggests HCC increases significantly within several hours following capture, a time too brief to be explained by this mechanism alone. In this study with captive grizzly bears, we sought to determine if a brief spike in blood cortisol concentration, thus mimicking a single stressful event, would cause an increase in HCC over a 7‐day period. To do this, we administered a single intravenous dose (5 μg/kg) of cosyntropin to three captive unanaesthetised adult female grizzly bears on two occasions, during April when hair growth was arrested and during August when hair was growing. In both trials, the cosyntropin caused a two‐fold or greater increase in serum cortisol levels within 1 hr but did not appear to influence HCC at 1, 48, and 168 hr following cosyntropin administration. We conclude the cosyntropin‐induced cortisol spike was likely insignificant when compared to the adrenocortical response that occurs in free‐ranging bears when captured. We suggest further study with a larger sample of captive bears to evaluate the combined effects of anaesthesia and multiple doses of cosyntropin administered over several hours would better simulate the adrenocortical response of free‐ranging grizzly bears during capture.

## Graphical abstract

Administration of cosyntropin to captive grizzly bears caused increased serum cortisol levels within 1 hr. However, cosyntropin did not influence hair cortisol concentration at 1, 48, and 168 hr following administration. Cosyntropin‐induced hair cortisol levels were insignificant compared to free‐ranging bears during capture.

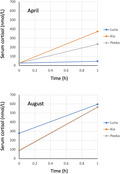

## 1 INTRODUCTION

Measuring cortisol deposited in hair is being used increasingly to evaluate long‐term stress in captive, free‐ranging, and domestic mammals. Various studies have evaluated the hair cortisol concentration (HCC) in response to, or in association with, potential long‐term stressors including relocation (Yamanashi et al., 2016), captivity (Carlitz et al., 2014), parasitism (Carlsson et al., 2016), anthropogenic disturbance (Wilson et al., 2020), and climate change (Bechshøft et al., 2013).

Other studies have emphasized the need to concurrently consider the potentially confounding effects of non‐stress factors on HCC including sex and age of animal (Terwissen et al., 2013), anatomical site of hair collection (Macbeth et al., 2010), method of hair collection (Sergiel et al., 2020), storage time of collected hair prior to hormone analysis (Azevedo et al., 2019), and method of hormone analysis (Kroshko et al., 2017). Different and sometimes conflicting findings among studies suggest further validation work, including the development of standardized protocols, is needed to confidently interpret cortisol levels in hair (Koren et al., 2018).

Fewer studies have questioned the evidence to support HCC as a biomarker of long‐term stress (Kalliokoski et al., 2019; Russell et al., 2014; Sharpley et al., 2009). Nonetheless, this is a fundamental question that appears to have been largely overlooked in the wide desire to apply a novel biomarker. This may also reflect an implicit assumption by many that the cortisol measured in hair was derived from the adrenal glands and subsequently sequestered from the bloodstream into growing hair. It follows that if the HCC is directly linked to the function of the hypothalamic‐pituitary‐adrenal (HPA) axis over an extended period, it must be a measure of long‐term stress. However, local production of cortisol within skin, including its epidermal and dermal compartments, as well as hair follicles, has been firmly established (Ito et al., 2005; Slominski & Mihm, 1996; Slominski et al., 2008) and shown to respond to stressors independent of the central HPA axis (Keckeis et al., 2012; Salaberger et al., 2016). Further, the rate of cortisol sequestration into hair from these two sources, central and local, is unclear (Keckeis et al., 2012) as is the influence of different types of stressors and their temporal characteristics, i.e., acute or chronic, intermittent or sustained (Heimbürge et al., 2019; Sharpley et al., 2012). Taken together, the degree to which the HCC reflects the central stress response of an individual (and, therefore, long‐term stress) remains uncertain (Kalliokoski et al., 2019).

Over the past decade, we have carried out numerous validation studies to determine if HCC can be used as a reliable indicator of long‐term stress in grizzly bears (*Ursus arctos*; Cattet et al., 2014; Kroshko et al., 2017; Macbeth et al., 2010; Sergiel et al., 2020). An important element of this research, the combination of controlled studies with captive grizzly bears and field studies with free‐ranging grizzly bears, has provided unique insight toward understanding what factors influence HCC in this species (Cattet et al., 2017, 2018). Although our understanding has broadened, we remain uncertain of the utility of HCC as an indicator of long‐term stress in live‐captured grizzly bears because of two consistent findings. One is the HCC is significantly lower in hair samples collected noninvasively by barbed wire snag than in hair samples collected from live‐captured bears (Cattet et al., 2014). The other is this difference in HCC is evident irrespective of whether hair growth is arrested (i.e., during spring) or active (i.e., during late summer and fall; Cattet et al., 2014). These findings suggest the HCC may increase rapidly over a duration of several hours or less in response to capture and, therefore, challenge the widely‐accepted hypothesis that cortisol in hair is primarily derived by passive diffusion from circulating blood into active hair follicles with its gradual accumulation as the hair grows (Russell et al., 2012; Short et al., 2016).

In this study with captive grizzly bears, we sought to determine if a brief spike in the cortisol concentration of the systemic blood circulation, thus mimicking a single stressful event, would cause an increase in HCC at several time points within 7 days on two occasions, during April when hair growth was arrested and during August when hair was growing. On both occasions, we challenged three bears, who were trained for blood sampling without anaesthesia, with a single intravenous dose of cosyntropin, a synthetic peptide that is identical to the 24‐amino acid segment at the N‐terminal of adrenocorticotropic hormone (ACTH) and exhibits the same corticosteroidogenic activity as natural ACTH (Hamilton & Cotton, 2010). Our intent with this design was to rule out the central stress response as being the primary cause of the high HCC values often recorded in free‐ranging grizzly bears within 24 hr following capture.

## 2 MATERIALS AND METHODS

We conducted the study during April and August 2015 with three adult female grizzly bears (Luna—12 years, Kio and Peeka—both 10 years) housed in captivity. These study bears and 15 other grizzly bears (nine females and six males) were housed in pairs in dens (3 m × 3 m × 2.5 m) with continuous access to an adjacent outdoor pen (3 m × 5 m × 5 m). During the active season (March–October), bears were released daily for 6–12 hr into an adjacent 0.56 ha outdoor enclosure.

The study bears had been previously trained using positive reinforcement techniques to enter a holding crate and present a rear leg though the bars of the crate for blood collection. Blood cortisol levels have been shown to be unaffected by this method (Joyce‐Zuniga et al., 2016).

To prevent exceeding the capacity of the facility with unwanted cubs, all adult female bears were subject to a birth control program that involved the oral administration of megestrol acetate (Ovaban^®^; Schering‐Plough Animal Health) at 80 mg/day from March 19–25, followed by the subcutaneous implantation of deslorelin acetate (Suprelorin^®^ 9.4 mg implant; Peptech Animal Health/Virbac) on April 3rd, 1 week prior to the first challenge.

We challenged each bear with cosyntropin (Cortrosyn^®^ ‐ Amphastar Pharmaceuticals, Inc.) by intravenous administration without anaesthesia on two occasions in 2015. Without any reference available for a cosyntropin dosage that would be appropriate for grizzly bears, we used the standard dosage (5 μg/kg) used for detecting ACTH deficiency in dogs (Scott‐Moncrieff, 2015). The first challenge occurred during April and the second during August. Our rationale for conducting challenges during these months was because HCC was reported to be affected by capture irrespective of whether bears were captured during spring when hair growth was arrested or during summer‐fall when hair was growing (Cattet et al., 2014).

To establish baseline cortisol concentrations in serum and hair, we collected a blood sample (10 ml) from the dorsal metatarsal or lateral saphenous vein into a serum tube and a hair sample (35 mg) from the shoulder immediately before cosyntropin administration.

After injecting cosyntropin, we collected blood at 1 hr (h), and hair at 1, 48 and 168 hr, post‐injection. All blood samples were centrifuged within 2 hr of collection and stored at −80°C until analysed.

To minimize variation in HCC because of differences in the anatomical site of collection (Macbeth et al., 2010), all hair samples were collected by shaving as close to the skin as possible from the same shoulder of each bear with each sampling site being distinct but in close proximity (i.e., ≤5 cm) to the site of the baseline sample. Hair samples were placed into paper envelopes using forceps. The envelopes were left open for several hours to ensure the samples were air‐dried, and then sealed and stored under low light at room temperature (20°C) until the time of laboratory analysis.

Serum cortisol concentrations were determined in duplicate using a commercial radioimmunoassay (RIA) kit (Corti‐cote RIA Kit; MP Biomedicals, LLC.). The assay detection limit was 4.69 nmol/L (Joyce‐Zuniga et al., 2016).

Hair samples were decontaminated, and cortisol was extracted from 25 mg subsamples, in accordance with an established protocol (Macbeth et al., 2010). Cortisol concentrations were determined in duplicate using a commercial ELISA kit (Oxford Biomedical; pre‐2016 EA65 kit). The approximate limit of detection for this kit was determined to be 0.32 pg of cortisol per mg of hair (Macbeth et al., 2010).

We conducted the cortisol assays for all serum and hair samples at the same time following the second challenge to prevent the confounding of results by variation between assays.

Given the small sample of animals used in this study, we limited our analysis to identifying trends in graphical presentations of the data.

## 3 RESULTS

**FIGURE 1:**
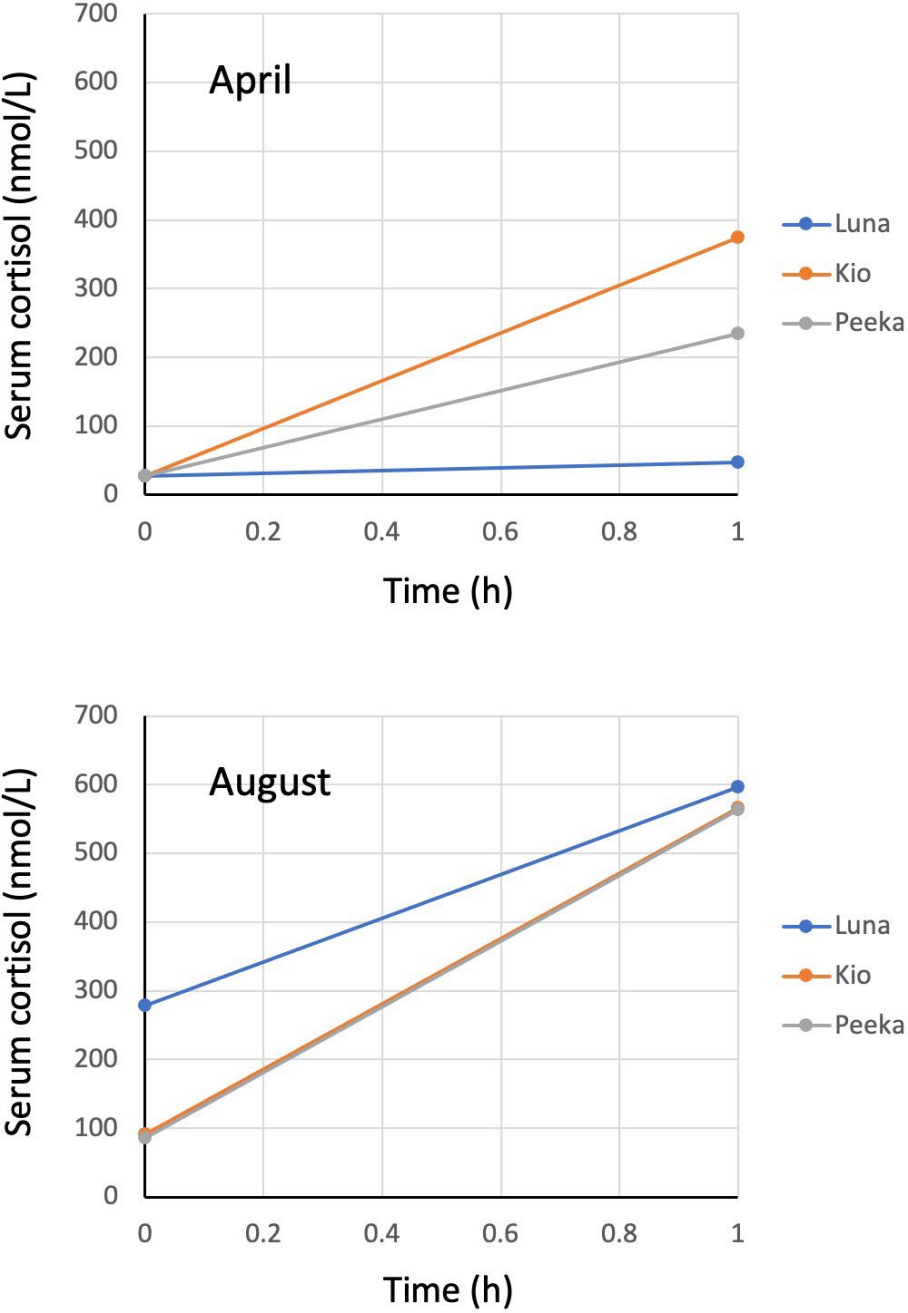
Serum cortisol concentrations of three adult female grizzly bears in April and August before and 1 hr following an intravenous injection of 5 μg/kg of cosyntropin. Note the lines for Kio and Peeka are overlapped in the August trial

Across the six trials (3 bears × 2 trials per bear), serum cortisol concentrations increased from a baseline average of 90 nmol/L (range: 28–279 nmol/L) to 397 nmol/L (47–596 nmol/L) at 1 hr following cosyntropin administration (Figure 1). The magnitude of increase varied markedly between bears with cortisol levels increasing no more than two‐fold with Luna in both trials but increasing five‐ to eight‐fold in the trials with Kio and Peeka.

**FIGURE 2:**
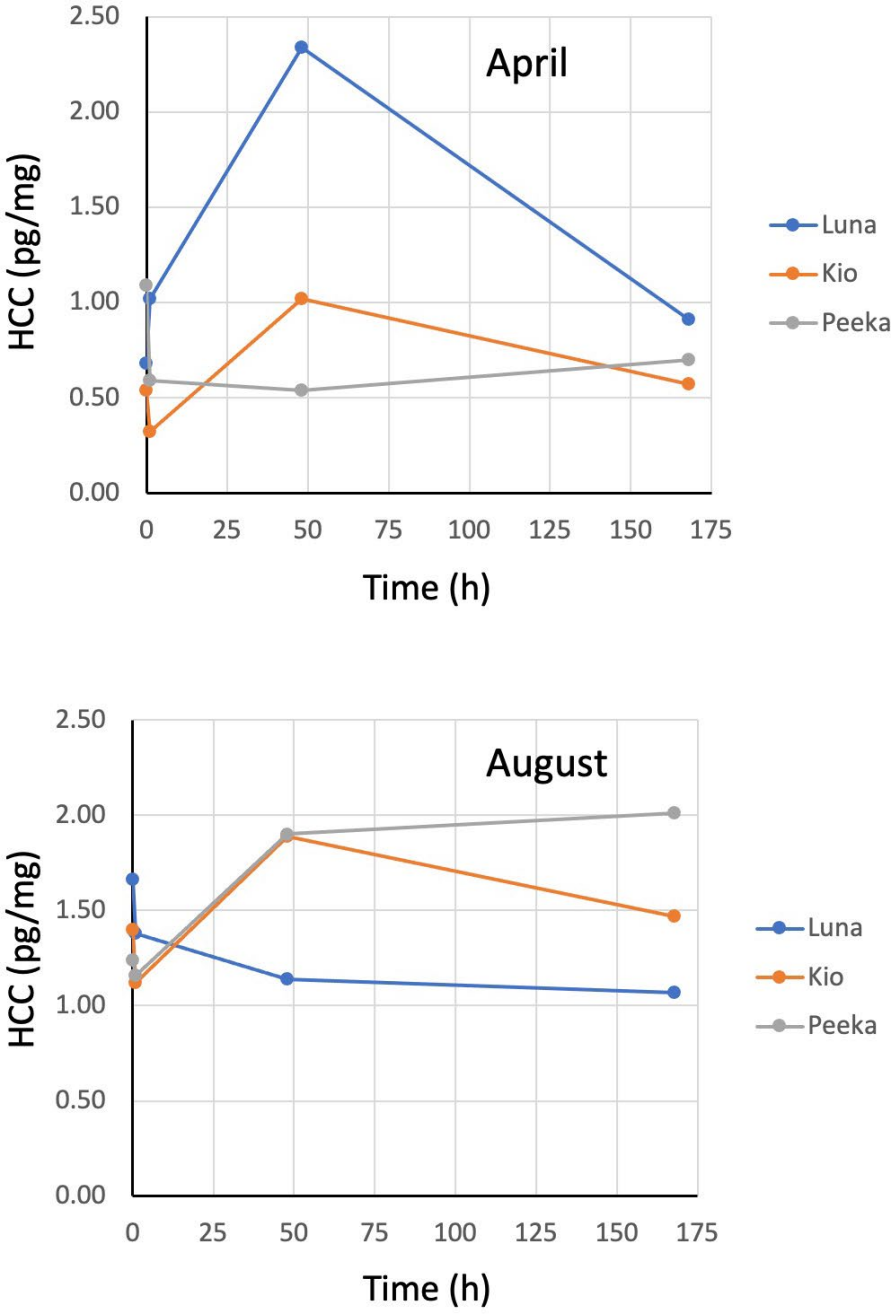
Hair cortisol concentrations (HCC) of three adult female grizzly bears in April and August before and 1, 48, and 168 hr following an intravenous injection of 5 μg/kg of cosyntropin

**FIGURE 3:**
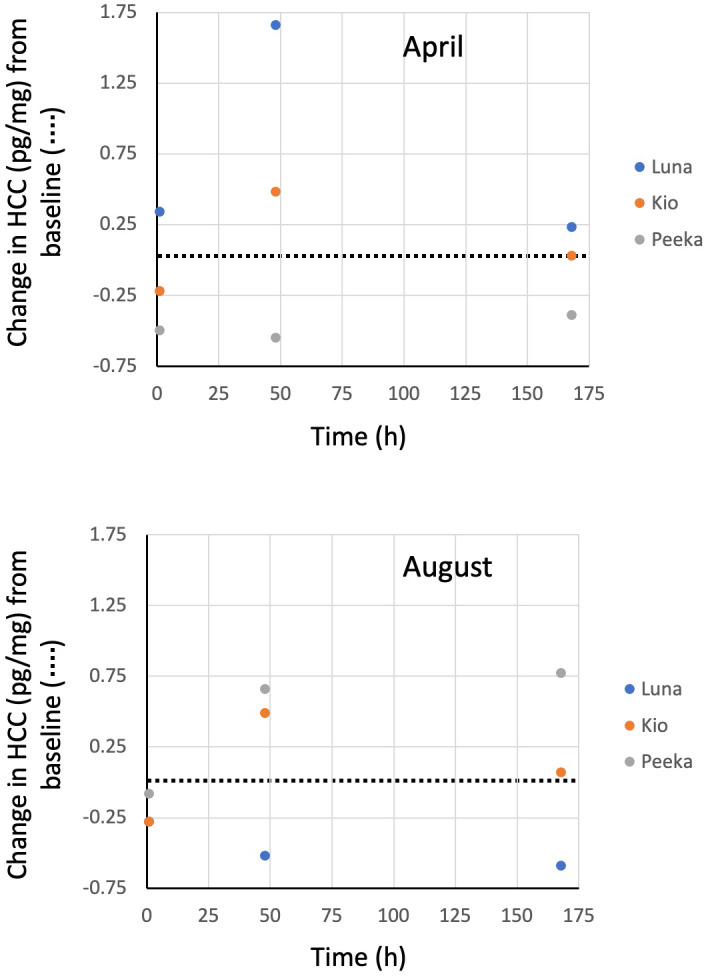
Changes in hair cortisol concentrations (HCC) relative to baseline values of three adult female grizzly bears in April and August at 1, 48, and 168 hr following an intravenous injection of 5 μg/kg of cosyntropin

Hair cortisol concentrations also changed from baseline concentrations across the six trials, but without any apparent trend (Figure 2). At 1 hr following cosyntropin administration, HCC decreased an average of −0.18 pg/mg (range: −0.50 to +0.34 pg/mg) from a mean baseline concentration of 1.10 pg/mg (Figure 3). At 48 hr, HCC increased an average of +0.37 pg/mg (−0.55 to +1.66 pg/mg) from baseline. And, at 168 hr, HCC increased an average of +0.02 pg/mg (−0.59 to 0.77 pg/mg) from baseline.

Baseline concentrations of cortisol were greater in both serum and hair in August (means: 152 nmol/L and 1.43 pg/mg) than in April (means: 28 nmol/L and 0.77 pg/mg; Figures 1 and 2).

## 4 DISCUSSION

To our knowledge this study is the first to evaluate cortisol levels in the serum and hair of grizzly bears following administration of synthetic ACTH. Despite observations from live‐captured grizzly bears suggesting HCC may change rapidly, we found no evidence in the present study to suggest HCC levels changed in response to a single dose (5 μg/kg) of cosyntropin. Nonetheless, this dose was sufficient to cause a marked increase in serum cortisol concentration within 1 hr.

In human subjects, serum cortisol levels usually peak at 45–60 min after cosyntropin injection, and a normal response is considered to be an approximate doubling of the baseline cortisol concentration (Hamilton & Cotton, 2010). With the exception of Luna's 1‐hr serum concentration in April (47 nmol/L; a 1.7‐fold increase from baseline), the 1‐hr values following all other challenges were at least twice the baseline value, and generally exceeded mean serum cortisol concentrations recorded for free‐ranging bears captured by remote drug delivery from helicopter (147 nmol/L, *n* = 41) and leg‐hold snare (222 nmol/L, *n* = 49; Cattet et al., 2003). Baseline serum cortisol concentrations in this study were greater on average when compared to values recorded in a previous study (means: 28 nmol/ml vs. 5 nmol/L) that used the same study animals and same procedures for blood collection (Joyce‐Zuniga et al., 2016), but this could have been caused by differences between studies in the timing of blood collection, i.e., morning versus night and first week of April versus last week of May. It is noteworthy, however, that researchers in the previous study also collected blood samples from four additional captive bears that were chemically immobilized by blow‐dart injection, and the serum cortisol concentrations for these bears (mean: 221 nmol/L) were similar to what we have recorded for free‐ranging bears following capture (Cattet et al., 2003). Despite differences in baseline cortisol concentrations between studies, we believe the dose of cosyntropin used in this study was sufficient to elicit a normal adrenocortical response. However, we recognize this response may not compare in intensity to the adrenocortical response that occurs in free‐ranging bears during and following capture. In the capture situation, serum cortisol is likely to be elevated for a longer duration, and the effect of sympathetic nervous system activation on cortisol release from the adrenal glands and its subsequent uptake into tissues is likely to be heightened.

Although the HCC of the three bears in this study appeared to change in an inconsistent manner following cosyntropin injection, we believe the differences between time points were largely due to the normal variation that occurs in cortisol concentration between individual hairs at any point in time rather than changes over time in response to the ACTH challenge. In a previous study, HCC values measured every 3 days over an 18‐day period in subsamples of hair collected from a live‐captured grizzly bear were found to vary by as much as ±0.81 pg/mg between time points (Macbeth et al., 2010). In this study, we found differences in HCC between baseline and post‐injection time points for the individual bears were within this range with only one of 18 values exceeding 0.81 pg/mg. Further, in our research with free‐ranging grizzly bears, we have observed HCC values in live‐captured bears to be significantly greater than values recorded for bears sampled without capture (median: 4.24 pg/mg, *n* = 137 live‐captured bears vs. 0.94 pg/mg, *n* = 303 bears sampled by barbwire snag; Cattet et al., 2014). In light of these previous studies, we believe it unlikely that the spike in serum cortisol concentration caused by a single intravenous dose of cosyntropin in the current study was sufficient to increase the HCC. The differences we observed between time points were more likely caused by the normal variation that occurs in cortisol concentration between individual hairs at any point in time.

The HCC response to an ACTH challenge has been evaluated in several other mammals. Dairy cattle (*Bos taurus*) treated three times with ACTH over a 2‐week period showed significantly higher HCC than saline‐treated or control animals (González‐de‐la‐Vara et al., 2011). Comparable results were found after three or more ACTH challenges over periods ranging from 2 weeks to more than 2 months in Canada lynx (*Lynx Canadensis*; Terwissen et al., 2013), eastern chipmunks (*Tamias striatus*; Mastromonaco et al., 2014), and mountain goats (*Oreamnos americanus*; Dulude‐de Broin et al., 2019). In contrast, single doses of ACTH were insufficient to increase HCC in barren ground caribou (*Rangifer tarandus granti*) and reindeer (*R. t. tarandus*; Ashley et al., 2011). Similarly, dairy cattle challenged twice with ACTH, with 1 week between challenges, did not show an increase in HCC (Tallo‐Parra et al., 2017). Collectively, these studies suggest hair cortisol may be insensitive to brief or non‐recurrent episodes of stress but responsive to prolonged or repeated stressful events. It follows that further study of HCC in grizzly bears should evaluate the cumulative effect of multiple ACTH challenges over a duration that is representative of the amount of time that may pass between the capture and sampling of a free‐ranging bear, e.g., ≤24 hr.

In the present study, cortisol concentrations in serum and hair tended to be greater during August than April. The serum results contrast with the findings of a previous study, done with the same study animals at the same facility, that showed cortisol levels to be greater during spring (March) than late summer (August) with cortisol levels decreasing as daylength increased (Ware et al., 2013). Similarly, the HCC results contrast with previous findings, again done with the same study animals at the same facility, where cortisol levels were found to be greater in April than August, although this difference was not statistically significant (Cattet et al., 2017). We propose these contrasting findings were caused by the birth control program that was in effect at the time of the April challenge trial in this study, but not in the previous studies. Specifically, megestrol acetate is known to markedly suppress the secretion of ACTH and cortisol in humans (Bodenner et al., 2005; Naing et al., 1999), and was recently demonstrated to elicit similar effects in captive bottlenose dolphins (*Tursiops truncates*; Houser et al., 2017). Further, these suppressive effects may persist for weeks to months following the discontinuation of megestrol acetate treatment (Houser et al., 2017; Middleton et al., 1987). In this study with captive grizzly bears, megestrol acetate treatment was completed approximately 2 weeks prior to the April challenge and, because of its persistent effects, could also account for the low serum and hair cortisol levels relative to values recorded following the August challenge.

From a broad perspective, this study adds to the knowledge of limitations and considerations in the use of HCC to evaluate long‐term stress in grizzly bears. While a single intravenous dose (5 μg/kg) of synthetic ACTH was sufficient to cause a marked increase in the cortisol concentration of the systemic blood circulation within 1 hr, it was not enough to increase the concentration of cortisol in hair for at least 1 week following administration. This would suggest the central stress response may not be the primary cause of the high HCC values often recorded in free‐ranging grizzly bears within 24 hr following capture. However, the challenge trials conducted in this study were likely insignificant when compared to the adrenocortical response that occurs in free‐ranging bears during and following capture. Some understanding for why the HCC of free‐ranging grizzly bears is generally high following capture could be gleaned from further studies with a larger sample of captive bears to evaluate the cumulative effect of multiple ACTH challenges over several hours and the effect of anaesthesia on HCC.

## AUTHOR CONTRIBUTION

**Marc Cattet:** Conceptualization; Data curation; Formal analysis; Methodology; Resources; Writing‐original draft. **David Janz:** Data curation; Resources; Software; Writing‐review & editing. **Lucy Kapronczai:** Data curation; Resources. **Joy Erlenbach:** Project administration; Resources; Writing‐review & editing. **Heiko Jansen:** Funding acquisition; Methodology; Project administration; Resources; Supervision; Writing‐review & editing. **O Lynne Nelson:** Methodology; Project administration; Resources; Writing‐review & editing. **Charles Robbins:** Funding acquisition; Investigation; Methodology; Project administration; Resources; Writing‐review & editing. **Gordon Stenhouse:** Conceptualization; Funding acquisition; Investigation; Resources; Supervision; Writing‐review & editing.

## ETHICAL STATEMENT

The authors confirm that the ethical policies of the journal, as noted on the journal's author guidelines page, have been adhered to and the appropriate ethical review committee approval has been received. We conducted the study during April and August 2015 with three adult female grizzly bears housed at the Washington State University Bear Research, Education, and Conservation Center. The bears had been previously trained using positive reinforcement techniques to enter a holding crate and present a rear leg though the bars of the crate for blood collection. The bears were maintained according to the Bear Care and Colony Health Standard Operating Procedures (Protocol #04873) based on U.S. Department of Agriculture guidelines. The project was approved by the Washington State University Institutional Animal Care and Use Committee (Protocol # 04636).

5

### PEER REVIEW

The peer review history for this article is available at https://publons.com/publon/10.1002/vms3.523.

## Data Availability

The datasets generated during and/or analysed during the current study are available from the corresponding author upon request.
